# Interrupted Time Series Analysis of Changes in Zolpidem Use Due to Media Broadcasts

**DOI:** 10.3390/ijerph18105114

**Published:** 2021-05-12

**Authors:** Bo-Ram Yang, Kyu-Nam Heo, Yun Mi Yu, Ga-Bin Yeom, Hye Duck Choi, Ju-Yeun Lee, Young-Mi Ah

**Affiliations:** 1 College of Pharmacy, Chungnam National University, Daejeon 34134, Korea; br.yang@cnu.ac.kr; 2 College of Pharmacy and Research Institute of Pharmaceutical Sciences, Seoul National University, Seoul 08826, Korea; bogopa8@snu.ac.kr; 3 Department of Pharmacy and Yonsei Institute of Pharmaceutical Sciences, College of Pharmacy, Yonsei University, Incheon 21983, Korea; yunmiyu@yonsei.ac.kr; 4 Department of Pharmaceutical Medicine and Regulatory Sciences, Colleges of Medicine and Pharmacy, Yonsei University, Incheon 21983, Korea; 5 College of Pharmacy, Yeungnam University, Gyeongsan 38541, Korea; iamgabin@naver.com (G.-B.Y.); choihd@yu.ac.kr (H.D.C.)

**Keywords:** zolpidem, broadcast, medication safety, interrupted time series analysis

## Abstract

Media has become a major source of information on health and plays a role in the decision-making process on health topics. We aimed to evaluate the association between zolpidem use and media broadcasts that reported the suicide risk. We obtained the data of adult outpatients who have been prescribed zolpidem or other hypnotics from the National Patient Sample database (2015–2017). We evaluated the change in zolpidem or other hypnotic prescription trends based on the prescription rate and average daily prescribed dose before and after July 2016, using interrupted time series analysis. A total of 129,787 adult patients had at least one zolpidem prescription in 3 years. The prescription rate of zolpidem after the broadcast decreased significantly by 0.178% (95% confidence interval (CI): −0.214, −0.142), whereas that of other hypnotic users did not differ from that before the broadcast (−0.020%, 95% CI: −0.088, 0.047). However, the trends in the prescription rate before and after the broadcast did not differ for zolpidem and other hypnotics. Broadcasting medication safety through major public media could have an effect on medication use. After broadcasting about the suicide risk of zolpidem, its overall prescription rate decreased immediately, but the trend was not changed.

## 1. Introduction

Zolpidem, a non-benzodiazepine sedative-hypnotic agent, was approved by the Korea Food and Drug Administration (KFDA) in 2005, and the use of zolpidem rapidly increased after approval [1]. Generally, zolpidem is considered safer than benzodiazepines because it has the following pharmacokinetic and pharmacodynamic properties; no active metabolites, shorter duration of action than benzodiazepines, selectivity for γ-aminobutyric acid type A receptor α1 subunits, and reduced dependence and withdrawal risks [2,3]. Therefore, zolpidem has been recommended as a pharmacological treatment for insomnia in clinical guidelines [4]. However, several serious adverse drug events, including hallucinations, memory impairment, vehicle-related accidents, and fractures, have been reported after zolpidem was marketed [5,6,7,8]. In addition, the risk of suicide after intaking zolpidem has been reported relatively recently in previous studies [9,10]. Sun et al. reported that the risk of suicide or suicide attempt increased approximately two-fold in zolpidem users (adjusted odds ratio (aOR) 2.08, 95% confidence interval (CI): 1.83, 2.36) [9]. A study in Korea also reported a similar increase in the risk of suicide among zolpidem users [11]. However, there have been no drug safety alerts from regulatory authorities related to suicide risk in patients using zolpidem in Korea. On the night of 16 July 2016, for about an hour, an in-depth exploration news broadcast by a major public media outlet raised the suspicion that the serial suicide of a celebrity family may be linked to zolpidem [12]. Following the broadcast, subsequent pieces of news, even across social media, conveyed the same content of the broadcast throughout July 2016. This broadcast also made accusations stating that other cases with abnormal shocking behavior (binge eating, violent behavior, and addiction) were also associated with zolpidem use. Moreover, the media held the physicians responsible for prescribing zolpidem without caution. Considering that suicide is a social issue in Korea, the broadcast might have had a huge impact on the general public [13]. When new safety issues related to severe adverse drug reactions are identified, the issues should be effectively and promptly propagated to healthcare providers (HCPs) and consumers to prevent fatal outcomes. Changes in labeling and packaging, safety alerts, patient information leaflets, educating the HCPs, patient screening or monitoring, and patient alert cards, have been used as methods of risk minimization activities [14]. Reducing unnecessary medications can be a major risk minimization strategy for drug safety management. Several studies have reported changes in medication use when various risk minimization methods are applied. The proportion of patients using low-dose zolpidem increased from 44% to 58% after a zolpidem label dose information change [15]. Kesselheim et al. also reported a 30% increase in the use of low-dose zolpidem, which was related to a drug safety communication (DSC) [16]. In a study by Touchard et al., zolpidem use decreased from 26% to 18.4% due to changes in the regulations on zolpidem [17]. In addition to these previous findings, a decrease in medication use by a drug utilization intervention program was also reported in previous studies [18,19].

Considering the nature of broadcasting, information is disseminated much faster through media than when using other risk minimization methods. In addition, media has become a major source of health information for the public; it is vital in forming individuals’ opinions and in their decision-making process on health-related topics [20]. Metlay et al. proposed that public media could be a tool to implement a direct-to-patient educational program on drug safety [21]. Moreover, the effects of mass media on utilizing health services and seeking medical advice have been evaluated [22,23]. However, there could be a publication bias in mass media because balanced information is generally un-newsworthy [24]. Additionally, the quality of lay media reporting medication safety has been known to be relatively poor [25]. Therefore, there are potential adverse effects related to health due to information provided by mass media. For example, Im et al. reported a negative correlation between the accuracy of patients’ belief about a medication and the exposure frequency to mass media regarding health information [26]. Furthermore, inappropriate information about vaccine safety via the media has also been associated with a low vaccination rate [27]. Considering the recent growth of social media, approximately three-quarters of internet users prefer social media as their source of health-related information; therefore, the impact of mass media on medication safety could be greater [28]. Therefore, risk communication for medications via media broadcasts may have a different pattern of effect on medication use compared with other risk minimization methods. However, most studies on media in health are related to advertising or analysis of the news itself [20,29]. Some studies have reported the effect of mass media as a channel of health information; however, most of them have simply evaluated the patient’s beliefs, medication adherence, or drug misuse through a survey conducted on patients [26,30].

To the best of our knowledge, no studies have evaluated the association between in-depth exploration news on drug safety by major public media and medication use. Therefore, in this study, we aimed to evaluate the influence of media broadcast on the suicide risk following zolpidem use by conduction an interrupted time series (ITS) study. 

## 2. Methods

In this study, we used sample databases from 2015 to 2017 provided by the Health Insurance Review and Assessment Service (HIRA), which annually extracts 3% of national patients using a stratified randomized sampling method. HIRA receives and evaluates claims from all medical institutions in Korea; thus, the HIRA databases include information on healthcare utilization, medication prescription, and basic demographic characteristics of 98% of the Korean population [31].

We selected adult outpatients (≥20 years) who were prescribed zolpidem or other hypnotics more than once from 2015 to 2017. We included patients using other hypnotics to compare the effect of broadcasting on medication use. Benzodiazepines are approved for insomnia (flunitrazepam, flurazepam, triazolam, and quazepam), alprazolam, which is commonly prescribed for off-label use in insomnia, and sedative antidepressants (trazodone, mirtazapine, and doxepin) were categorized as other hypnotics.

This study was approved by the Yeungnam University Institutional Review Board (YU 2019-01-001). The requirement for informed consent was waived owing to the retrospective nature of the study and the use of de-identified data from the HIRA database. 

To analyze the effect of broadcasting on prescription practice, we determined the prescription rate of medications included in this study (zolpidem or other hypnotics) in adult outpatients each month. We used the number of patients prescribed with the medications included in this study as the numerator and the number of adult patients who visited the medical institutions for ambulatory care as the denominator. We also evaluated the monthly, average daily prescribed dose per patient using the defined daily dose (DDD). 

Baseline characteristics such as age, sex, insurance, and comorbidities, including depression (International Classification of Disease, tenth Revision (ICD-10) codes F32, F33, and F341), bipolar disorder (ICD-10 codes F30, F31, F340, F348, and F349), anxiety (ICD-10 codes F40–F43), schizophrenia (ICD-10 codes F20–F29), substance use disorder (ICD-10 codes F10–F19), dementia (ICD-10 codes F00–F03), and headache (ICD-10 codes G43, G44, and R51) were identified as covariates. We also evaluated the Charlson comorbidity index (CCI) to identify multiple morbidities [32].

The famous, in-depth exploration news broadcast by a major public media outlet and subsequent news in July 2016 (intervention) may have affected (interrupted) the previously established trend in zolpidem use (prescription rate and average daily prescribed dose). Therefore, we used ITS analysis with segmented regression analyses to model changes in the levels and trends of zolpidem use associated with the broadcasting that was reported in this study. The following equation was used for the segmented regression model:

*Y*_t_ = *β*_0_ + *β*_1_ × time*_t_* + *β*_2_ × broadcast*_t_* + *β*_3_ × time after broadcast + *ε_t_*, (1)

where *Y*_t_ is the prescription rate or average daily prescribed dose in a month, *t* ranges from 1 to 36 months, *β*_0_ is the baseline intercept, *β*_1_ is the average monthly change in zolpidem use before broadcast, *β*_2_ is the immediate effect of the broadcast, *β*_3_ is the change in the slope of the monthly zolpidem use after the broadcast, and *ε_t_* is the error term.

Time, the number of months from the start of the study, ranges from 1 to 36 months, whereas broadcast is a dummy variable with 0 and 1 categories for the months before and after the broadcast, respectively. Time after the broadcast, a continuous variable indicating the number of months after the broadcast, was assigned as 0 before the broadcast. The before and after intervention periods occurred from January 2015 to June 2016 and from August 2016 to December 2017, respectively. We checked for autocorrelation using the Durbin–Watson statistic among the monthly prescription rates or average daily prescribed dose and corrected for this using a stepwise autoregression to select the order of the autoregressive error model where necessary.

To identify differences according to covariates, subgroup analysis was performed according to the following variables: sex, insurance, age group (20–39, 40–64, and ≥65 years), CCI score (0, 1 or 2, and ≥3), and comorbid disease (psychiatric disorder). 

Descriptive statistics, such as mean (standard deviation) and number (percentage), were calculated. Chi-square test was used to compare categorical variables, and Student’s *t*- test was used to compare continuous variables between the two groups. We used SAS version 9.4 (SAS Institute, Inc., Cary, NC, USA) for data management and statistical analysis. The significance level was set at *p* < 0.05.

## 3. Results

The annual ratio of outpatients using zolpidem decreased from 3.73% (42,921) in 2015 to 3.72% (43,505) in 2016 and to 3.69% (43,361) in 2017, whereas the use of other hypnotics increased from 6.77% (77,828) in 2015 to 6.92% (81,028) in 2016 and to 7.00% (81,192) in 2017 (*p*-value < 0.05). Almost half of the patients aged from 40 to <65 years were prescribed zolpidem (46.11%–48.35%) and other hypnotics (45.81%–47.29%) compared with those in other age groups, and more than half were female in both groups (62.89%–63.21% zolpidem users; 64.57%–65.15% other hypnotics users). Patients using other hypnotics were more frequently diagnosed with depression, anxiety disorder, schizophrenia, and headache than those prescribed zolpidem in the 3 years of study (*p* < 0.0001). The proportion of patients with dementia was also higher among other hypnotic users than zolpidem users; however, a higher proportion of zolpidem users had CCI scores ≥ 3 (Table 1).

**Figure 1:**
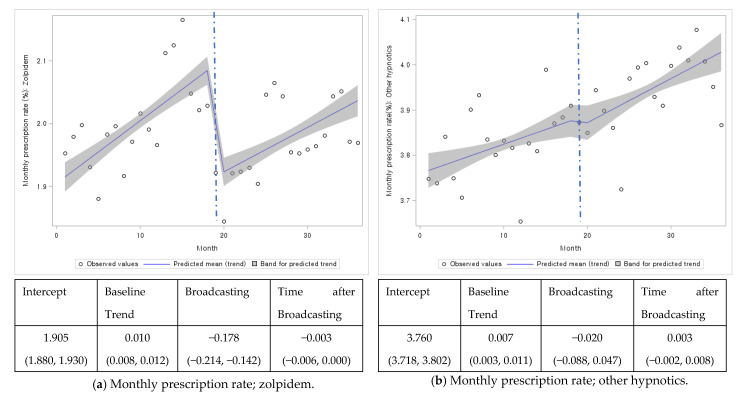

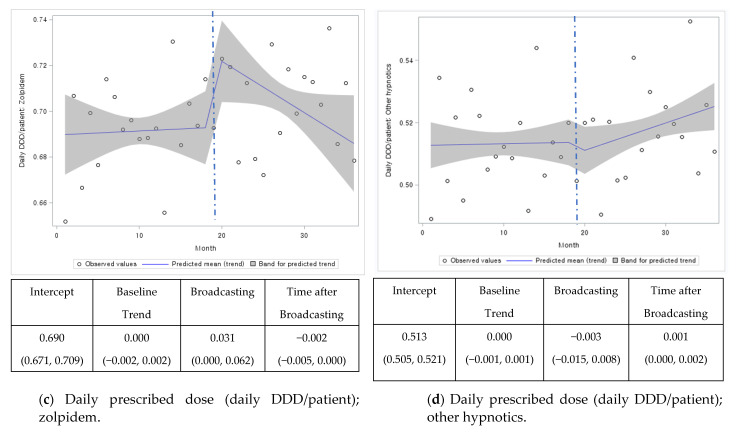
Overall monthly prescription rate or average daily prescribed dose of zolpidem or other hypnotics, 2015–2017. DDD; Defined daily dose.

The intercepts of zolpidem and other hypnotics were 1.905% and 3.760%, respectively. At the time of broadcast, the proportion of patients who were prescribed zolpidem decreased (−0.178%, 95% CI: −0.214%, −0.142%), whereas no significant change was observed in the proportion of patients using other hypnotics (−0.020%, 95% CI: −0.088%, 0.047%). However, changes in the trend of prescription rate after the broadcast for zolpidem (−0.003%, 95% CI: 0.006, 0.000%) or other hypnotics (0.003%, 95% CI; −0.002%, 0.008%) did not differ from those before the broadcast. Evaluation of the changes in the monthly, average daily prescribed doses showed that the trends during (0.031 DDD/patient/month, 95% CI; 0.000, 0.062) and after broadcast (−0.002 DDD/patient/month, 95% CI: −0.005, 0.000) were significantly different from before the broadcast (*p*-value < 0.05). However, the extent of the change was minimal. In other hypnotic users, there was no significant change in the trend of the average daily prescribed doses after (0.001 DDD/patient/month, 95% CI; 0.000, 0.002) and during the broadcast (−0.003 DDD/patient/month, 95% CI: −0.015, 0.008) (Figure 1).

In all subgroup analyses, we identified that the zolpidem prescription rate at broadcast was significantly lower than that before the broadcast. The extent of change at broadcast was higher in elderly patients (−0.239%, 95% CI: −0.367, −0.112), in medical aid beneficiaries (−0.391%, 95% CI: −0.538, −0.244), those with a national meritorious service award (−0.715%, 95% CI; −1.175, −0.255), patients with psychiatric disease (−0.528%, 95% CI: −0.663, −0.394), and those with high CCI score (−0.250%, 95% CI: −0.377, −0.123). The trend after the broadcast was not significantly different from before the broadcast in most subgroups. Even in the group showing a significant change in trend after the broadcast, the magnitude of the change was minimal (Table 2).

There was no meaningful change in the zolpidem average daily prescribed dose after and at broadcast. In most subgroups of other hypnotic users, the prescription rate and the average daily prescribed dose at and after the broadcast were not significantly different from before the broadcast. In some subgroups, such as medical aid users and those with national meritorious service awards, we identified a statistically significant change after the broadcast; however, the change was a very small one (Table 2 and Table 3).

## 4. Discussion

In this study, we found an effect of broadcasting on medication use using national claims data. The proportion of patients using zolpidem in the month of the broadcast was approximately 0.2% lower than that before the broadcast (1.9%). In other words, it indicates that almost 10% of patients who used zolpidem before the broadcast immediately discontinued the medication after the broadcast. Considering that there was no change in the proportion of patients using other hypnotics with similar pharmacological effects as zolpidem, the trend changes in zolpidem use in the month of broadcast could be explained by the effect of broadcasting on zolpidem safety. The change in medication use according to the spread of medication safety information is well known [15,33,34]. However, the sources of information include label change or DSC led by an authorization body such as the FDA; thus, the matter of obtaining safety information was mainly through HCPs. Meanwhile, since media broadcasts are accessible to an unspecified general population, their impact might be different. Compared with previous studies reporting an increase in low-dose zolpidem users according to DSC (30%, *p* < 0.001) or label change (14%, *p* = 0.002) [15,16], the extent of change in our study was lower and can be considered temporary. This could be explained by the difference in the reliability of the information provider, propagation method, and target audience. The low preference for news reports on medication information safety in a study by Kesselheim et al. supports these points [35]. In a previous study that compared participants who watched reality television programs for 1 h where illegal drug use and prescription drug misuse were mentioned with participants who did not watch such shows, a higher risk of illegal drug use (aOR 3.40, 95% CI 1.55, 7.46) was reported in participants who watched [36]. Therefore, the association between an in-depth broadcast by major public media and the change in zolpidem use in our study could be explained by the result of the aforementioned study. In addition, there was no label change or DSC about the use of zolpidem around July 2016, and other z-drugs (e.g., eszopiclone) were not approved in Korea during that time.

Unlike the change in the prescription rate of zolpidem, there was no clinically meaningful difference in the average daily prescribed dose of zolpidem at the month of the broadcast and after the broadcast compared with that before the broadcast. In other words, the broadcast did not have a significant effect on the decision on zolpidem dosage.

In the subgroup analysis, a trend similar to that of overall outpatients was observed; however, the extent of decrease in the prescription rate of zolpidem was greater in elderly patients, medical aid users, national meritorious service beneficiaries, patients with a psychiatric disorder, and those with high CCI score. This finding is related to the high prevalence of zolpidem use before the broadcast among these patients. The high prevalence of zolpidem use in this population is consistent with the prevalence of insomnia in vulnerable patient groups [37].

Our study had some limitations. First, we could not evaluate the change in the actual medication-taking behavior due to the characteristics of the claims data. However, considering that the claims data is widely used in studies on medication adherence, the change in prescription trends could reflect the patients’ medication-taking behavior. Second, we could not categorize zolpidem use into incidence or prevalence groups because we used annual claims data. Third, some medications included in the other hypnotics group might not have been used as hypnotics. However, the trend change in other hypnotics between psychiatric and non-psychiatric patients was not different. Fourth, it was impossible to directly identify the risk factors for changes in medication use because of the study design, although we identified trend changes according to subgroups. Lastly, we could not evaluate the direct influence of broadcast on the prescriber, although physicians often obtained information on medication safety through online sources [38], and we did not analyze the content of the broadcast. To our knowledge, there were no other explainable factors; however, it cannot be ruled out that unknown factors other than the media broadcast might have influenced zolpidem use.

This study was the first to report changes in medication use related to medication safety information by broadcasting, not by propagating medication safety information generated by regulatory authorities. It is not uncommon that official information about medication safety, such as DSC, might not reach physicians or patients [39]; thus, dissemination of medication safety information through the media could be a potential complementary measure of risk communication. In addition, there is a need for studies evaluating the impact of media broadcast as a risk communication method because previous studies have been limited in this aspect of media coverage for medication safety information announced by the regulatory authorities [40].

## 5. Conclusions

In this study, based on an analysis of the national claims database, we identified the effect of information on medication safety disseminated by major public media, which contained information revolving around suspicions related to suicides in a celebrity family rather than being based on scientific evidence. However, the effect was immediate and transient. Further research is required on the impact of media broadcasts on health outcomes, including harm reduction and disease control, on changes in medication use. Moreover, broadcasters must be more cautious and responsible while providing information related to medication, considering the identified influence of media broadcast in this study.

## Figures and Tables

**Table 1:** Baseline characteristics of patients who used zolpidem or other hypnotics as outpatients from 2015 to 2017.

	Zolpidem (*n* = 129,787)						Other Hypnotics (*n* = 241,048)					
	2015		2016		2017		2015		2016		2017	
	*n*	(%)	*n*	(%)	*n*	(%)	*n*	(%)	*n*	(%)	*n*	(%)
Number of user	42,921	(3.73)	43,505	(3.72)	43,361	(3.69)	77,828	(6.77)	81,028	(6.92)	82,192	(7.00)
Age ^a,b,c^												
20–39	5864	(13.66)	5766	(13.25)	5311	(12.25)	12,204	(15.68)	13,096	(16.16)	13,133	(15.98)
40–64	19,793	(46.11)	20,232	(46.50)	20,964	(48.35)	35,871	(46.09)	37,121	(45.81)	38,867	(47.29)
65+	17,264	(40.22)	17,507	(40.24)	17,086	(39.40)	29,753	(38.23)	30,811	(38.03)	30,192	(36.73)
Sex, Female ^a,b,c^	27,132	(63.21)	27,359	(62.89)	27,278	(62.91)	50,707	(65.15)	52,316	(64.57)	53,130	(64.64)
Type of health insurance ^a,b,c^												
Health Insurance	38,666	(90.09)	39,088	(89.85)	38,891	(89.69)	70,364	(90.41)	73,370	(90.55)	74,195	(90.27)
Medical Aid	3892	(9.07)	4063	(9.34)	4138	(9.54)	6986	(8.98)	7176	(8.86)	7478	(9.10)
National Meritorious service	363	(0.85)	354	(0.81)	332	(0.77)	478	(0.61)	482	(0.59)	519	(0.63)
Comorbidities												
Depression ^a,b,c^	13,678	(31.87)	13,739	(31.58)	13,279	(30.62)	31,181	(40.06)	32,513	(40.13)	33,547	(40.82)
Bipolar disorder	2932	(6.83)	2874	(6.61)	3086	(7.12)	5215	(6.70)	5484	(6.77)	6181	(7.52)
Anxiety disorder ^a,b,c^	16,804	(39.15)	16,773	(38.55)	16,714	(38.55)	47,206	(60.65)	49,058	(60.54)	50,174	(61.04)
Schizophrenia ^b,c^	1827	(4.26)	1718	(3.95)	1766	(4.07)	3482	(4.47)	3536	(4.36)	3957	(4.81)
Substance Use Disorder ^a^	1314	(3.06)	1188	(2.73)	1328	(3.06)	2210	(2.84)	2161	(2.67)	2542	(3.09)
Headache ^a,b,c^	13,409	(31.24)	12,900	(29.65)	12,977	(29.93)	27,796	(35.71)	28,184	(34.78)	28,711	(34.93)
Dementia ^a,b,c^	2993	(6.97)	3092	(7.11)	3030	(6.99)	5720	(7.35)	6106	(7.54)	6304	(7.67)
CCI score, mean ± SD ^a,b,c^	2.33	±2.36	2.42	±2.40	2.26	±2.23	2.18	±2.20	2.27	±2.27	2.10	±2.11
0	9672	(22.53)	9231	(21.22)	9723	(22.42)	18,210	(23.40)	18,062	(22.29)	19,701	(23.97)
1 or 2	17,584	(40.97)	17,776	(40.86)	18,177	(41.92)	33,086	(42.51)	34,104	(42.09)	35,316	(42.97)
≥ 3	15,665	(36.50)	16,498	(37.92)	15,461	(35.66)	26,532	(34.09)	28,862	(35.62)	27,175	(33.06)

^a,b,c^: *p*-values for chi-square test or *t*-test (Charlson Comorbidity Index (CCI) score) between zolpidem users and other hypnotic users in 2015(a), 2016(b), and 2017(c) were less than 0.05.

**Table 2:** Subgroup analysis showing changes in the prescription rate before and after broadcasting. (unit: patients using the study medications/total outpatients monthly, percentage).

Subgroup	Zolpidem Estimate (95% CI; Lower, Upper)				Other Hypnotics Estimate (95% CI; Lower, Upper)			
	Intercept	Baseline Trend	Broadcasting	Time after Broadcasting	Intercept	Baseline Trend	Broadcasting	Time after Broadcasting
Overall	1.905 (1.880, 1.930)	0.010 (0.008, 0.012)	**−0.178** **(−0.214, −0.142)**	−0.003 (−0.006, <0.001)	3.760 (3.718, 3.802)	0.007 (0.003, 0.011)	−0.020 (−0.088, 0.047)	0.003 (−0.002, 0.008)
Male	1.649 (1.616, 1.682)	0.01 (0.008, 0.012)	**−0.172** **(−0.202, −0.142)**	**−0.004** **(−0.007, −0.001)**	3.067 (2.996, 3.137)	0.006 (0.000, 0.013)	−0.022 (−0.123, 0.080)	−0.001 (−0.010, 0.009)
Female	2.119 (2.017, 2.221)	0.008 (0.001, 0.015)	**−0.170** **(−0.266, −0.074)**	0.000 (−0.010, 0.010)	4.280 (4.191, 4.369)	0.009 (0.000, 0.017)	−0.033 (−0.164, 0.098)	0.000 (−0.012, 0.012)
Age group								
20–39	0.820 (0.801, 0.839)	0.008 (0.006, 0.009)	**−0.133** **(−0.162, −0.104)**	**−0.003** **(−0.006, −0.001)**	1.615 (1.568, 1.662)	0.012 (0.008, 0.017)	0.027 (−0.045, 0.100)	0.001 (−0.006, 0.006)
40–64	1.741 (1.715, 1.768)	0.010 (0.008, 0.012)	**−0.170** **(−0.201, −0.14)**	**−0.003** **(−0.006, 0.000)**	3.450 (3.392, 3.508)	0.004 (0.000, 0.008)	−0.051 (−0.112, 0.009)	0.004 (−0.002, 0.010)
65+	3.382 (3.302, 3.462)	0.009 (0.001, 0.017)	**−0.239** **(−0.367, −0.112)**	0.003 (−0.006, 0.013)	6.553 (6.471, 6.635)	0.009 (0.004, 0.014)	−0.047 (−0.118, 0.023)	0.000 (−0.007, 0.008)
Health insurance	1.733 (1.706, 1.761)	0.008 (0.006, 0.011)	**−0.164** **(−0.201, −0.127)**	−0.003 (−0.006, 0.001)	3.393 (3.319, 3.467)	0.006 (−0.001, 0.013)	−0.008 (−0.115, 0.098)	0.000 (−0.011, 0.010)
Medical Aid	5.329 (5.238, 5.421)	0.045 (0.037, 0.054)	**−0.391** **(−0.538, −0.244)**	−0.003 (−0.014, 0.009)	11.542 (11.387, 11.697)	0.019 (0.005, 0.034)	−0.192 (−0.415, 0.031)	**0.052** **(0.031, 0.074)**
National Meritorious service	4.866 (4.581, 5.151)	0.012 (−0.016, 0.039)	**−0.715** **(−1.175, −0.255)**	0.029 (−0.008, 0.065)	6.580 (6.231, 6.928)	0.003 (−0.027, 0.032)	−0.157 (−0.568, 0.253)	**0.069** **(0.024, 0.114)**
Psychiatric disorder (−)	0.844 (0.819, 0.870)	0.007 (0.005, 0.009)	**−0.102** **(−0.137, −0.067)**	−0.002 (−0.006, 0.001)	0.670 (0.651, 0.690)	0.002 (0.000, 0.003)	−0.014 (−0.033, 0.005)	0.001 (−0.001, 0.003)
Psychiatric disorder ^(1)^ (+)	6.451 (6.343, 6.559)	0.038 (0.029, 0.047)	**−0.528** **(−0.663, −0.394)**	**−0.022** **(−0.035, −0.009)**	17.024 (16.759, 17.290)	0.071 (0.046, 0.095)	−0.086 (−0.468, 0.296)	−0.029 (−0.065, 0.007)
CCI score								
0	0.984 (0.958, 1.010)	0.007 (0.004, 0.009)	**−0.100** **(−0.143, −0.057)**	−0.001 (−0.004, 0.002)	2.004 (1.960, 2.047)	0.005 (0.001, 0.009)	0.009 (−0.061, 0.079)	**0.010** **(0.005, 0.016)**
1 and 2	1.873 (1.814, 1.933)	0.005 (0.001, 0.01)	**−0.166** **(−0.235, −0.097)**	0.003 (−0.004, 0.01)	3.776 (3.671, 3.881)	−0.004 (−0.013, 0.006)	−0.015 (−0.162, 0.133)	**0.020** **(0.005, 0.034)**
≥3	3.862 (3.781, 3.942)	0.003 (−0.004, 0.011)	**−0.250** **(−0.377, −0.123)**	**0.015** **(0.005, 0.025)**	7.162 (7.052, 7.273)	0.006 (−0.005, 0.016)	−0.021 (−0.180, 0.138)	−0.001 (−0.016, 0.015)

^(1)^ Psychiatric disorders; depression, bipolar disorder, anxiety, schizophrenia, and substance use disorder. Bold: *p*-value < 0.05 for broadcasting and time after broadcasting.

**Table 3:** Subgroup analysis for showing changes in the prescription rate before and after broadcasting. (unit: daily DDD/patient, monthly).

Subgroup	Zolpidem Estimate (95% CI; Lower, Upper);				Other Hypnotics Estimate (95% CI; Lower, Upper)			
	Intercept	Baseline Trend	Broadcasting	Time after Broadcasting	Intercept	Baseline Trend	Broadcasting	Time after Broadcasting
Overall	0.690 (0.671, 0.709)	0.000 (−0.002, 0.002)	**0.031** **(0.000, 0.062)**	**−0.002** **(−0.005, 0.000)**	0.513. (0.505, 0.521)	0.000 (−0.001, 0.001)	−0.003 (−0.015, 0.008)	0.001 (0.000, 0.002)
Male	0.703 (0.700, 0.706)	0.001 (0.001, 0.001)	0.003 (−0.001, 0.007)	**−0.001** **(−0.001, −0.001)**	0.550 (0.54, 0.560)	0.000 (−0.001, 0.001)	0.002 (−0.012, 0.016)	0.001 (0.002, 0.003)
Female	0.669 (0.653, 0.686)	0.001 (0.000, 0.003)	0.000 (−0.023, 0.023)	−0.001 (−0.003, 0.001)	0.492 (0.4, 0.494)	0.000 (0.000, 0.001)	**−0.006** **(−0.009, −0.002)**	**0.001** **(0.000, 0.001)**
Age group								
20–39	0.654 (0.635, 0.672)	0.002 (0.001, 0.004)	0.004 (−0.023, 0.031)	**−0.003** **(−0.005, 0.000)**	0.491 (0.475, 0.508)	0.002 (0.000, 0.003)	−0.009 (−0.033, 0.015)	−0.001 (−0.004, 0.001)
40–64	0.670 (0.659, 0.681)	0.001 (0.000, 0.002)	0.004 (−0.012, 0.019)	−0.001 (−0.002, 0.001)	0.534 (0.526, 0.542)	0.001 (0.000, 0.002)	−0.001 (−0.013, 0.011)	−0.001 (−0.002, 0.001)
65+	0.707 (0.691, 0.722)	0.000 (−0.001, 0.001)	0.000 (−0.012, 0.013)	0.000 (−0.001, 0.001)	0.496 (0.487, 0.506)	−0.001 (−0.002, 0.000)	−0.004 (−0.018, 0.009)	**0.002** **(0.001, 0.003)**
Health insurance	0.668 (0.659, 0.678)	0.001 (0.000, 0.001)	0.002 (−0.008, 0.012)	−0.001 (−0.002, 0.000)	0.481 (0.473, 0.488)	0.000 (0.000, 0.001)	−0.006 (−0.016, 0.005)	0.001 (0.000, 0.002)
Medical Aid	0.7782 (0.7642, 0.7922)	0.0009 (−0.0004, 0.0023)	0.0055 (−0.0154, 0.0264)	−0.0011 (−0.003, 0.0009)	0.69 (0.686, 0.693)	0.000 (0.000, 0.000)	−0.001 (−0.006, 0.003)	0.000 (−0.001, 0.001)
National Meritorious service	0.8831 (0.7576, 1.0086)	0.011 (−0.001, 0.023)	0.0544 (−0.1496, 0.2583)	**−0.0226** **(−0.038, −0.0073)**	0.737 (0.555, 0.919)	−0.001 (−0.006, 0.003)	**0.137** **(0.077, 0.197)**	0.001 (−0.005, 0.007)
Psychiatric disorder (−)	0.572 (0.554, 0.590)	0.002 (0.000, 0.004)	0.000 (−0.026, 0.025)	−0.002 (−0.005, 0.000)	0.319 (0.309, 0.328)	0.001 (0.000, 0.001)	−0.007 (−0.015, 0.001)	**0.001** **(0.000, 0.002)**
Psychiatric disorder ^(1)^ (+)	0.746 (0.734, 0.758)	0.001 (−0.001, 0.002)	−0.001 (−0.018, 0.016)	0.000 (−0.002, 0.002)	0.546 (0.543, 0.549)	0.000 (0.000, 0.000)	−0.002 (−0.006, 0.003)	**0.001** **(0.000, 0.001)**
CCI score								
0	0.650 (0.631, 0.669)	0.002 (0.000, 0.004)	0.005 (−0.022, 0.032)	**−0.003** **(−0.005, 0.000)**	0.514 (0.505, 0.524)	0.000 (−0.001, 0.001)	0.003 (−0.010, 0.016)	0.000 (−0.001, 0.002)
1 and 2	0.669 (0.659, 0.679)	0.000 (0.000, 0.001)	0.004 (−0.006, 0.015)	0.000 (−0.001, 0.001)	0.499 (0.492, 0.506)	0.000 (−0.001, 0.001)	−0.004 (−0.014, 0.006)	**0.001** **(0.000, 0.002)**
≥3	0.717 (0.702, 0.732)	0.001 (0.000, 0.002)	0.001 (−0.013, 0.015)	−0.001 (−0.002, 0.001)	0.526 (0.523, 0.529)	0.000 (0.000, 0.000)	**−0.006** **(−0.009, −0.002)**	**0.001** **(0.001, 0.001)**

^(1)^ Psychiatric disorders; depression, bipolar disorder, anxiety, schizophrenia, and substance use disorder. Bold: *p*-value < 0.05 for broadcasting and time after broadcasting.

## Data Availability

We used the national sample data provided by the Health Insurance Review and Assessment Service (HIRA) in South Korea. Other researchers can request access to the data directly from the HIRA System via instruction at the following URL: https://opendata.hira.or.kr/op/opc/selectPatDataAplInfoView.do (accessed on 14 March 2021).
